# Prediction model for the risk of osteoporosis incorporating factors of disease history and living habits in physical examination of population in Chongqing, Southwest China: based on artificial neural network

**DOI:** 10.1186/s12889-021-11002-5

**Published:** 2021-05-26

**Authors:** Yuqi Wang, Liangxu Wang, Yanli Sun, Miao Wu, Yingjie Ma, Lingping Yang, Chun Meng, Li Zhong, Mohammad Arman Hossain, Bin Peng

**Affiliations:** 1 Department of Epidemiology and Health Statistics, School of Public Health and Management, Chongqing Medical University, Chongqing, 400016 China; 2 School of Basic Medicine, Kunming Medical University, Kunming, 650031 China; 3 The First Affiliated Hospital of Chongqing Medical University Health Management Center, Chongqing, 400016 China; 4 The First Affiliated Hospital of Chongqing Medical University, Department of Urology, Chongqing, 400016 China

**Keywords:** Osteoporosis, Disease history, Living habits, Prediction model, Artificial neural network, Physical examination

## Abstract

**Background:**

Osteoporosis is a gradually recognized health problem with risks related to disease history and living habits. This study aims to establish the optimal prediction model by comparing the performance of four prediction models that incorporated disease history and living habits in predicting the risk of Osteoporosis in Chongqing adults.

**Methods:**

We conduct a cross-sectional survey with convenience sampling in this study. We use a questionnaire From January 2019 to December 2019 to collect data on disease history and adults’ living habits who got dual-energy X-ray absorptiometry. We established the prediction models of osteoporosis in three steps. Firstly, we performed feature selection to identify risk factors related to osteoporosis. Secondly, the qualified participants were randomly divided into a training set and a test set in the ratio of 7:3. Then the prediction models of osteoporosis were established based on Artificial Neural Network (ANN), Deep Belief Network (DBN), Support Vector Machine (SVM) and combinatorial heuristic method (Genetic Algorithm - Decision Tree (GA-DT)). Finally, we compared the prediction models’ performance through accuracy, sensitivity, specificity, and the area under the receiver operating characteristic curve (AUC) to select the optimal prediction model.

**Results:**

The univariate logistic model found that taking calcium tablet (odds ratio [OR] = 0.431), SBP (OR = 1.010), fracture (OR = 1.796), coronary heart disease (OR = 4.299), drinking alcohol (OR = 1.835), physical exercise (OR = 0.747) and other factors were related to the risk of osteoporosis. The AUCs of the training set and test set of the prediction models based on ANN, DBN, SVM and GA-DT were 0.901, 0.762; 0.622, 0.618; 0.698, 0.627; 0.744, 0.724, respectively. After evaluating four prediction models’ performance, we selected a three-layer back propagation neural network (BPNN) with 18, 4, and 1 neuron in the input layer, hidden and output layers respectively, as the optimal prediction model. When the probability was greater than 0.330, osteoporosis would occur.

**Conclusions:**

Compared with DBN, SVM and GA-DT, the established ANN model had the best prediction ability and can be used to predict the risk of osteoporosis in physical examination of the Chongqing population. The model needs to be further improved through large sample research.

**Supplementary Information:**

The online version contains supplementary material available at 10.1186/s12889-021-11002-5.

## Background

Osteoporosis is defined by the World Health Organization (WHO) as a ‘skeletal disease characterized by low bone mass and microarchitectural deterioration of bone tissue’ [1]. About 200 million people worldwide suffer from osteoporosis, while around 88 million people in China suffer from it [2]. With increasing age, the incidence of osteoporotic fractures increases, and these fractures significantly reduce the quality of life. Moreover, osteoporosis is usually detected when a fracture occurs. Fortunately, osteoporosis can be prevented. Research by Yu et al. revealed that improving lifestyle can control and prevent osteoporosis [3]. For example, it has been found that taking calcium tablet, cooking, and doing housework were associated with lowering the risk of osteoporosis, while smoking, drinking alcohol, and sedentary behavior were positively correlated with osteoporosis. The research results also revealed that certain diseases were related to osteoporosis [4]. People with a history of hyperthyroidism, hypertension, coronary heart disease (CHD), diabetes mellitus (DM), and other diseases had a higher risk of osteoporosis.

Because the risk factors of osteoporosis interacted each other by a non-linear mechanism, it was difficult for traditional linear regression models and logistic regression models to solve collinearity [5, 6]. Therefore, machine learning approaches, combinatorial heuristics, and other specific algorithms may be required [7]. In addition, machine learning approaches were the new method in the field of public health. However, few studies assessed risk factors for osteoporosis based on these models [8]. Therefore, this study aims to illustrate the potential use of ANN in predicting the risk of osteoporosis by comparing the performance of four prediction models that combined with disease history and living habits.

## Methods

### Study subjects

**Fig. 1:**
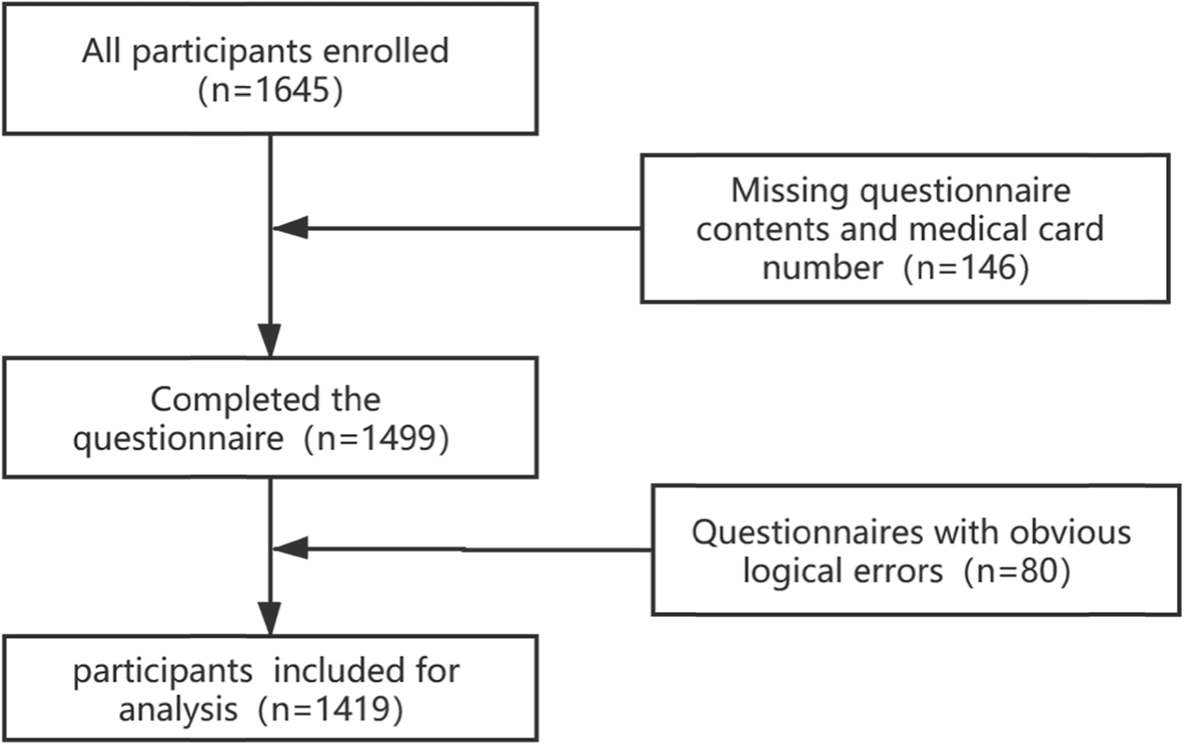
The inclusion process of participants in this study

Considering the availability of physical examination data and questionnaire data as well as the operability of the study, this study selected a convenience sampling to distribute personal health status and lifestyle questionnaire designed by the research team (see more in Supplementary File 1) to the adults who got dual-energy X-ray absorptiometry in the Medical Examination Center of the First Affiliated Hospital of Chongqing Medical University. Participants who had previously been diagnosed with osteoporosis were excluded. Questionnaires with missing content, especially those with missing medical card numbers and obvious logical errors will be excluded from the analysis. In the end, 1419 questionnaires (all over 20 years old) were effectively returned, and the effective response rate was 86.2%. The inclusion process of participants in this study is shown in Fig. 1.

### Data collection

Well-trained staff conducted a questionnaire survey on all subjects to obtain their information. The questionnaire consists of three parts, general information (including name, gender, medical card number), personal health (including hyperthyroidism, hypertension, CHD, DM, chronic gastrointestinal disease, chronic renal failure, gout, and malignant tumor), and living habits (take calcium tablet, smoking status, drinking status, cooking status, the nature of work, the main mode of transportation to work, housework, and physical exercise). Physical examination (weight, height, waist circumference (WC), blood pressure) was measured with standard medical equipment. The participants’ bone mineral density (BMD) was measured using DXA (MEDIX DR 2D-fan beam densitometer; Medilink, France). Daily phantom scans were performed every morning for quality control, and all BMD scans were conducted by well-trained examiners using standardized procedures, according to the recommended protocols by manufacturers. Body mass index (BMI) was calculated as weight in kilograms divided by the square of height in meters (kg/m^2^).

### Definitions

Normal BMD was defined if the T-score was more than − 1.0, osteopenia was defined if the T-score was less than or equal to − 1.0 and more than − 2.5, and osteoporosis was defined if the T-score was less than or equal to − 2.5, according to the WHO criteria (1994) [9]. As osteoporosis and osteopenia were the similar entity in nature, and the number of osteoporosis cases were small in this study, osteoporosis and osteopenia were combined into one outcome, which is called low BMD [10]. A history of hyperthyroidism, hypertension, CHD, DM, chronic gastrointestinal disease, chronic renal failure, gout, and malignant tumor was defined as received treatment or taking medication. Drinking was dichotomized (current drinker vs former or nondrinker). Smoking was also dichotomized (current smoker vs former or nonsmoker). The questionnaire defined cooking as cooking more than five times a week; Housework as never, occasionally, often; Calcium tablets as taking it for more than seven consecutive days. The nature of work is divided into three levels: sedentary, mild labor, manual labor. Sedentary behaviors have been classified into a range of human endeavors that result in an energy expenditure of no more than 1.5 times’ resting energy expenditure [11]. Manual labor was mainly based on physical activity, which required strong physical strength, greater muscle contraction, and physical movement of the limbs in space [12]. Mild labor falls somewhere between this sedentary and manual labor. The main modes of transportation to work are divided into four categories: working from home, walking, taking the bus and driving. Physical exercise was defined as whether they have participated in physical exercise lasting more than 20 min each time in recent years. There were two possible answers - yes and no.

### Statistical analysis

Baseline characteristics were statistically described as mean ± standard deviation (SD). Continuous variables were analyzed by *analysis of variance* (ANOVA), and categorical variables were tested by *chi-squared* test. The univariate logistic regression analysis was used to estimate odds ratios (ORs) and 95% confidence intervals (CIs) of baseline characteristics related to osteoporosis. We used SAS statistical software (version 9.4; SAS Institute Inc., Cary, North Carolina) to analyze the data.

All eligible participants (*N* = 1419) were randomly divided into two sets: the training set (N1 = 993) and the test set (N2 = 426) in the ratio of 7:3, the process was based on deep learning of ANN for proportional division [13, 14]. The input variables of osteoporosis prediction model based on ANN, DBN, SVM were selected by univariate logistic model (*P* < 0.20) [15], and the input variables of osteoporosis prediction model based on combination heuristic were selected by GA-DT. These models required that all variable values be standardized to 0 to 1. Binary variables were divided into 0 and 1; Non-binary variables were standardized to the range of 0 to 1 by the formula: ${X}_m=\frac{X_m-{X}_{min}}{X_{max}-{X}_{min}}$ . After four prediction models were established, we compared the risk assessment models’ performance through accuracy, sensitivity, specificity, and receiver operating characteristic curve (ROC) to select the optimal prediction model. ROC curve is a visualized optimal model, which shows the performance of binary classifiers by considering the sensitivity, specificity, and accuracy of the model [16, 17]. Unless specified, we used the significance level of 0.05 for all analyses. Use software R version 4.0.2 (R Foundation for Statistical Computing, Vienna, Austria) and MATLAB (version R2013b; MathWorks.Inc., U.S.A) to build prediction models.

## Results

### Characteristics of subjects

Table 1 summarized the baseline characteristics of the normal BMD and the low BMD in all study populations. In total study population, 460 out of 1419 subjects had low BMD, while the other 959 were normal. Compared with the normal BMD group, the low BMD group subjects were older and had lower BMI (*P* < 0.001). In the low BMD group, the incidence of fracture was higher (10.64% vs. 17.61%) and CHD (0.94% vs. 3.91%), the difference was statistically significant (*P* < 0.001), but other diseases such as hyperthyroidism, DM, chronic gastrointestinal disease, chronic renal failure, gout, and malignant tumor showed no significant difference between the two groups. The two groups had statistically significant differences in drug use (such as calcium tablets and estrogen drugs) and living habits (such as smoking, drinking, cooking, the nature of work, the main mode of transportation to work, doing the household work and physical exercise). There were no significant differences between two groups in terms of diastolic blood pressure (DBP).

**Table 1:** ^a^ Baseline characteristics of the normal BMD and the low BMD in all study populations

	Overall (*N* = 1419)		
Characteristics	Normal BMD	Low BMD	*P-*value
Age (years)	49.03 ± 10.87	59.82 ± 8.59	< 0.0001
Height (cm)	163.08 ± 8.15	158.80 ± 7.66	< 0.0001
Weight (kg)	64.48 ± 11.25	58.91 ± 9.28	< 0.0001
BMI (kg/m^2^)	24.14 ± 3.11	23.31 ± 2.90	< 0.0001
SBP (mmHg)	124.76 ± 19.16	128.35 ± 19.19	0.0010
DBP (mmHg)	76.60 ± 11.96	76.64 ± 11.66	0.9474
Fracture	102 (10.64%)	81 (17.61%)	0.0002
Hyperthyroidism	20 (2.09%)	13 (2.83%)	0.3863
Hypertension	139 (14.49%)	90 (19.57%)	0.0151
CHD	9 (0.94%)	18 (3.91%)	0.0001
DM	58 (6.05%)	40 (8.70%)	0.0657
Chronic gastrointestinal disease	104 (10.84%)	61 (13.26%)	0.4097
Chronic renal failure	50 (5.21%)	25 (5.43%)	0.8617
Gout	31 (3.23%)	6 (1.30%)	0.0329
Malignant tumor	7 (0.73%)	4 (0.87%)	0.7789
Estrogen drugs	5 (0.52%)	11 (2.39%)	<.0001
Corticosteroids	3 (0.21)	1 (0.22%)	0.7510
Calcium tablet	167 (17.41%)	151 (32.83%)	<.0001
Smoking	233 (24.30%)	87 (18.91%)	0.0231
Drinking	371 (38.69%)	116 (25.22%)	<.0001
Cooking	456 (47.55%)	299 (65.00%)	<.0001
The nature of work			0.0003
sedentary	462 (48.18%)	169 (36.74%)	
light activity	455 (47.45%)	268 (58.26%)	
manual labor	42 (4.38%)	23 (5.00%)	
The main mode of transportation to work			<.0001
working at home	250 (26.07%)	128 (27.83%)	
walking	221 (23.04%)	78 (16.69%)	
taking buses	172 (17.94%)	197 (42.83%)	
driving	316 (32.95%)	57 (12.39%)	
Do the household work			<.0001
Never	101 (10.53%)	43 (9.35%)	
Occasionally	455 (47.45%)	143 (31.09%)	
Often	403 (42.02%)	274 (59.57)	
Physical exercise	635 (66.21%)	333 (72.39%)	0.0193

a: Values are Mean ± standard deviation, median (interquartile range) or number (percentage)

### Risk factors for osteoporosis

As shown in Table 2, the related risk factors of osteoporosis from using univariate logistic regression model based on all samples (*N* = 1419). From the univariate logistic regression model, we found significantly protective factors for osteoporosis: height (OR = 0.934, 95%CI:0.921–0.948), weight (0.950, 95%CI:0.939–0.961), BMI (OR = 0.913, 95%CI:0.879–0.948), take calcium tablet (OR = 0.431, 95%CI:0.334–0.558). Continuous physical exercise for 20 min a day is also a protective factor for osteoporosis (OR = 0.747, 95%CI:0.585–0.954). There were also risk factors: in terms of gender, women were more likely to suffer from osteoporosis than men (OR = 1.963, 95%CI:1.566–2.461). As we age, osteoporosis was more likely to occur, age (OR = 1.109, 95%CI = 1.094–1.124). SBP (OR = 1.010, 95%CI:1.004–1.015), history of fracture (OR = 1.796, 95%CI:1.309–2.462), CHD (OR = 4.299, 95%CI:1.916–9.644), current drinking (OR = 1.835, 95%CI:1.436–2.345).

**Table 2:** Analysis of risk factors of osteoporosis using univariate logistic regression model

Variable	*β*	Standard Error (SE)	*P-*value	Odds ratio	95% confidence interval
Female	0.675	0.115	< 0.001^**^	1.963	(1.566,2.461)
Age (years)	0.104	0.007	< 0.001^**^	1.109	(1.094,1.124)
Height (cm)	−0.068	0.008	< 0.001^**^	0.934	(0.921,0.948)
Weight (kg)	−0.051	0.006	< 0.001^**^	0.950	(0.939,0.961)
BMI (kg/m^2^)	−0.091	0.019	< 0.001^**^	0.913	(0.879,0.948)
SBP (mmHg)	0.010	0.003	0.001^**^	1.010	(1.004,1.015)
DBP (mmHg)	0.000	0.005	0.947	1.000	(0.991,1.010)
Medical history					
Fracture	0.585	0.161	< 0.001^**^	1.796	(1.309,2.462)
Hyperthyroidism	0.311	0.361	0.388	1.365	(0.673,2.770)
Hypertension	0.361	0.149	0.015^*^	1.435	(1.071,1.922)
CHD	1.458	0.412	< 0.001^**^	4.299	(1.916,9.644)
DM	0.392	0.214	0.067^*^	1.479	(0.973,2.250)
Chronic gastrointestinal disease	0.229	0.172	0.185^*^	1.257	(0.897,1.762)
Chronic renal failure	0.044	0.252	0.862	1.045	(0.638,1.711)
Gout	0.927	0.450	0.039^*^	2.528	(1.047,6.102)
Malignant tumor	0.176	0.629	0.799	1.193	(0.347,4.096)
Use of medication					
Corticosteroids	−0.365	1.156	0.752	0.694	(0.072,6.693)
Calcium tablet	−0.841	0.131	< 0.001^**^	0.431	(0.334,0.558)
Living habit					
Smoking	0.234	0.129	0.069^*^	1.264	(0.981,1.628)
Drinking	0.607	0.125	< 0.001^**^	1.835	(1.436,2.345)
Cooking	0.717	0.117	< 0.001^**^	2.049	(1.628,2.578)
The nature of work					
Sedentary	–	–	–	Ref.	1.00
Light activity	0.476	0.118	< 0.001^**^	1.610	(1.277,2.031)
Manual labor	0.403	0.275	0.142^*^	1.497	(0.874,2.564)
The main mode of transportation to work					
Working at home	0.805	0.151	< 0.001^**^	2.237	(1.665,3.006)
Walking	–	–	–	Ref.	1.00
Taking buses	−0.372	0.171	0.029^*^	0.689	(0.493,0.963)
Driving	−1.043	0.180	< 0.001^**^	0.352	(0.247,0.502)
Do the household work					
Never	–	–	–	Ref.	1.00
Occasionally	−0.304	0.206	0.140^*^	0.738	(0.493,1.105)
Often	0.468	0.198	0.018^*^	1.597	(1.083,2.355)
Physical exercise	−0.291	0.125	0.020^*^	0.747	(0.585,0.954)

*P < 0.2, **P < 0.05

### Establishment of the prediction models for osteoporosis

**Fig. 2:**
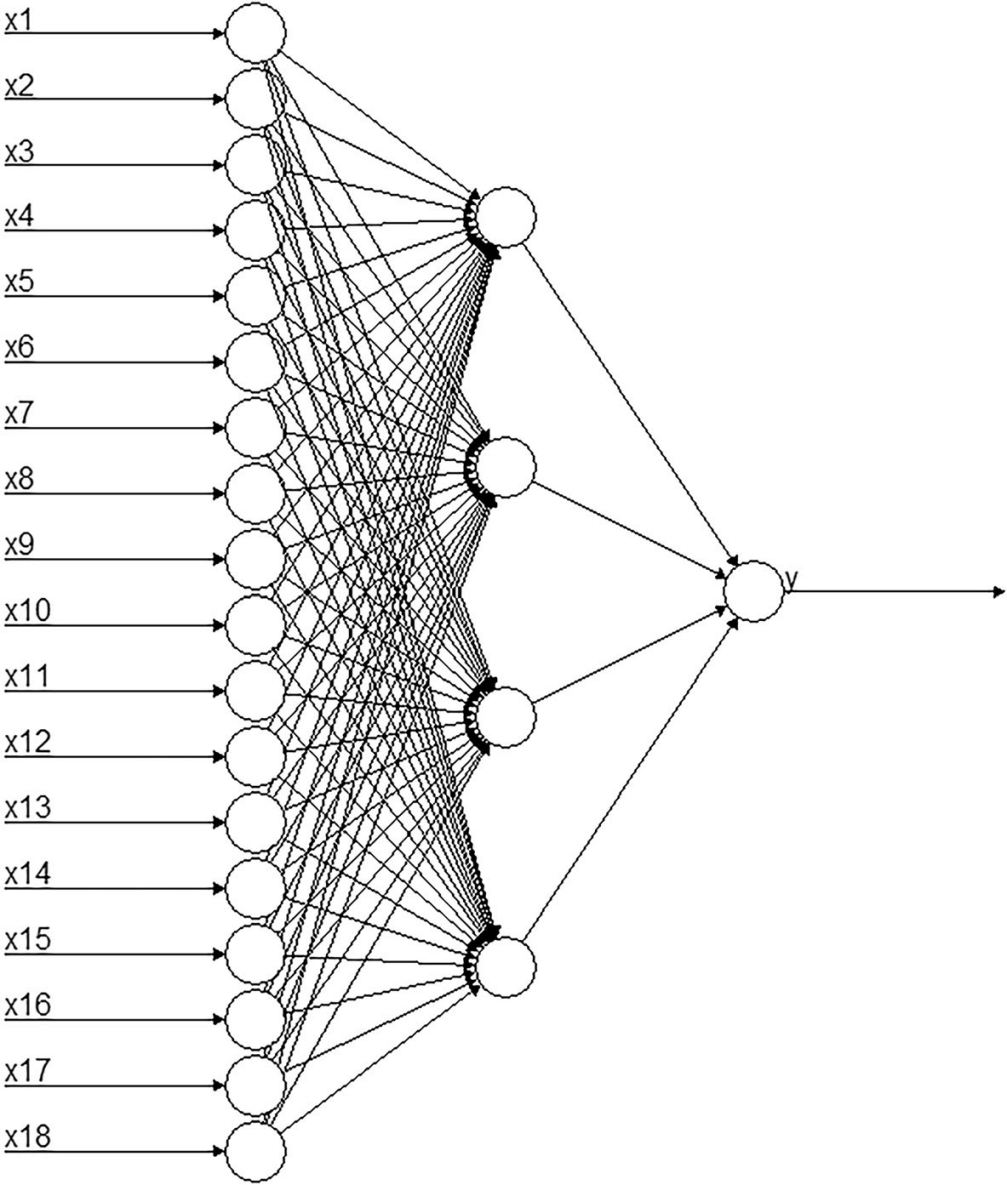
Graphic representation of the basic architecture of ANN used in training set. *x*_1_ represents age, *x*_2_ represents gender, *x*_3_ represents BMI, *x*_4_ represents SBP, *x*_5_ represents history of fracture, *x*_6_ represents history of hypertension, *x*_7_ represents history of CHD, *x*_8_ represents history of DM, *x*_9_ represents history of chronic gastrointestinal disease, *x*_10_ represents history of gout, *x*_11_ represents take calcium tablet, *x*_12_ represents cooking, *x*_13_ represents drinking alcohol, *x*_14_ represents smoking, *x*_15_ represents the nature of work, *x*_16_ represents the main mode of transportation to work, *x*_17_ represents do the household work, *x*_18_ represents physical exercise, and *y* represents osteoporosis

We established an ANN model based on factors related to osteoporosis derived from univariate logistic regression analysis. Gender, age, BMI, SBP, disease history (including fracture, hypertension, CHD, DM, chronic gastrointestinal disease, gout) and living habits (including taking calcium tablet, smoking, drinking, cooking, the nature of work, the main mode of transportation to work, doing the housework, physical exercise) as the input layer of the model. The output variable was a binary variable of whether a person had osteoporosis. The ANN structure consisted of three layers (Fig. 2), and the parameters were selected according to previous related studies [6, 18]. Training parameters such as learning rate and momentum were set at their default values. The training function was based on the back propagation (BP) with momentum algorithm. The neural network was trained for 46,673 steps. Training parameters such as learning rate and momentum of the DBN model have also default values. Through model debugging, the optimal DBN model was six neurons in the hidden layer. The prediction model based on GA-DT included 13 input variables.

### The performance of four prediction models

**Fig. 3:**
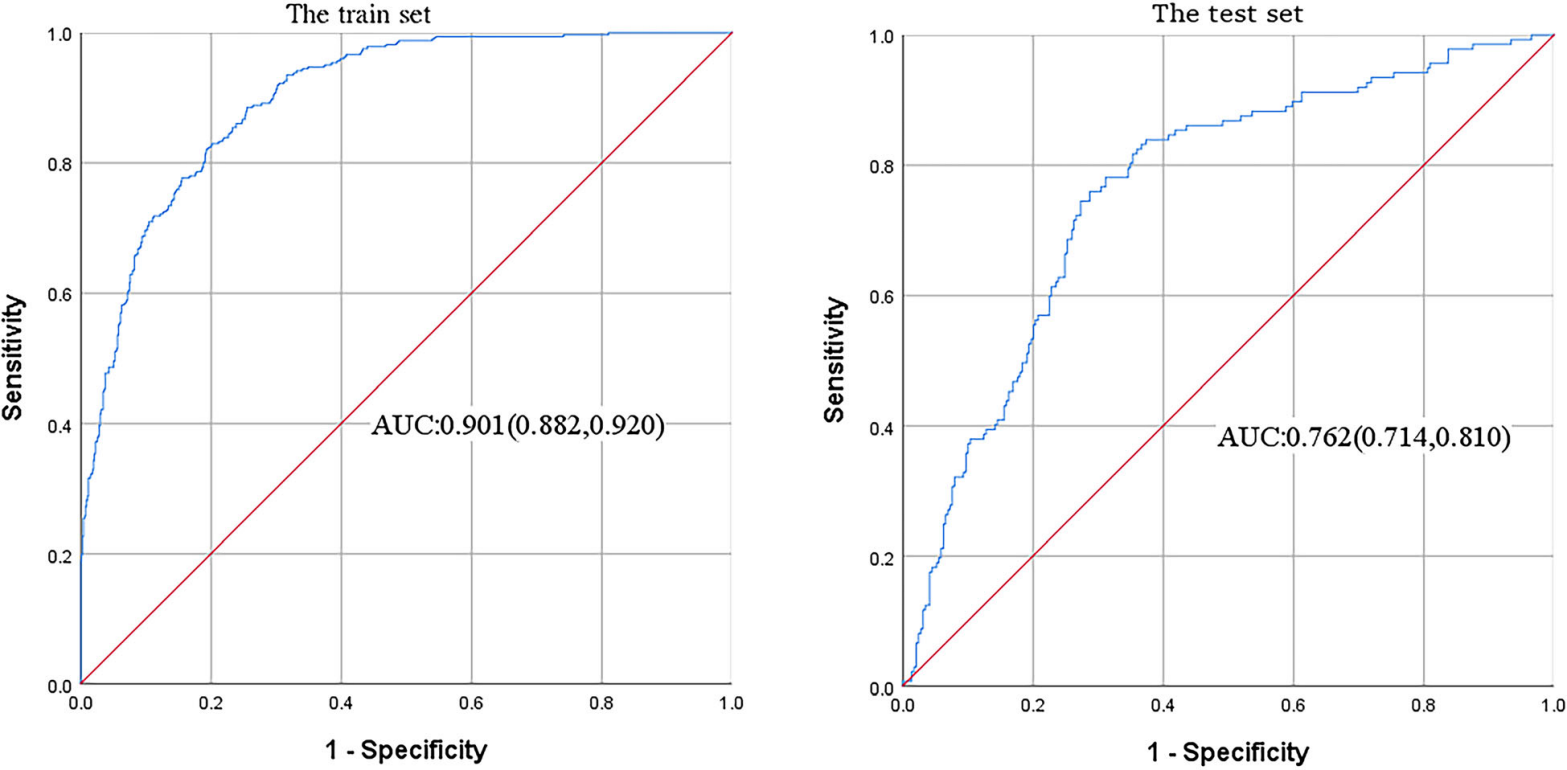
The receiver operating characteristic (ROC) curves obtained from the ANN in training set and test set

Table 3 compared four prediction models through accuracy, sensitivity, specificity, AUC. The four indicators of the ANN model were better than the DBN. SVM and GA-DT had higher sensitivity than the ANN model, but the specificity was extremely low. Figure 3 shown the AUC obtained from the training set and test set of the ANN model. The AUC of the training set was 0.901, and the AUC of the test set was 0.762. The AUC of the training set and the test set of the prediction model based on DBN, SVM, and GA-DT were 0.622, 0.618; 0.698, 0.627; 0.744, 0.724, respectively. Therefore, the well-trained ANN model had the highest accuracy and AUC, and can accurately predict the risk of an individual suffering from osteoporosis.

**Table 3:** The performance of four prediction models on training set and test set

Model	Data set	Accuracy	Sensitivity	Specificity	AUC (95% CI)
ANN	Training set	0.801	0.833	0.785	0.901 (0.882,0.920)
	Test set	0.728	0.708	0.737	0.762 (0.714,0.810)
DBN	Training set	0.634	0.517	0.689	0.622 (0.585,0.659)
	Test set	0.629	0.496	0.695	0.618 (0.561,0.675)
SVM	Training set	0.772	0.920	0.492	0.698 (0.661,0.758)
	Test set	0.725	0.888	0.340	0.627 (0.588,0.625)
GA-DT	Training set	0.778	0.840	0.649	0.744 (0.710,0.779)
	Test set	0.763	0.849	0.585	0.724 (0.689,0.760)

## Discussion

This was a novel study to develop an osteoporosis prediction model based on disease history and living habits. Our study established an osteoporosis prediction models based on ANN with momentum algorithm, DBN, SVM, and GA-DT. Through the comparison of four prediction models, in addition to AUC, the accuracy, sensitivity, and specificity also indicated that the ANN model we constructed was reliable. For the training set and the test set, the AUCs of ANN model were 0.901 and 0.762, respectively. For the training group and test group, the cut-off probability of osteoporosis was 0.330. That is, when the probability of occurrence was significant than 0.330, osteoporosis will occur. In clinical diagnosis, ANN had great value. According to the relevant factors of the patient, the probability value of each patient was calculated. In 2017, Song et al. [19] used BP neural network, decision tree model and logistic regression model to predict the risk of individual DM. Through the AUC value, the BP neural network had the best performance in predicting the risk of DM.

ANN is essentially a mathematical model, and its structure is similar to biological neural network. The input layer is the first layer in the ANN. It takes input signals (values) and passes them on to the next layer. A hidden layer in an ANN is a layer in between input layers and output layers, where artificial neurons take in a set of weighted inputs and produce an output through an activation function. It is a typical part of nearly any neural network in which engineers simulate the types of activity that go on in the human brain. The output layer in an ANN is the last layer of neurons that produces given outputs for the program [20]. The ANN models have been widely applied in research because they can model highly non-linear systems in which the relationship among the variables is unknown or very complicated, especially in medical field, which is an emerging phenomenon [21]. In most study cases [22–26], the ANN model is of practical value for predicting the 5-year overall survival rate after gastrectomy for gastric cancer, diagnosing congenital heart disease in pregnant women, and analyzing the risk of thyroid cancer and monitoring the trends and the incidence of AIDS in China and the classification of leukemia.

ANN includes the BP algorithm and BP with momentum algorithm. The BP algorithm is widely regarded as a powerful tool for training feedforward neural networks. However, because it uses the steepest descent method to update the weights, its convergence speed is prolonged, and it often obtains sub-optimal solutions [27]. On the contrary, the BP of the momentum algorithm converges quickly. The significance of this model is that the probability of suffering from osteoporosis can be obtained when age, gender, BMI, SBP, history of fracture, history of hypertension, history of CHD, history of DM, history of chronic gastrointestinal disease, history of gout, taking calcium tablet, cooking, drinking alcohol, smoking, the nature of work, the main mode of transportation to work, doing the housework, and physical exercise were given as input into the model. These input variables are easily available. This model can be used as a tool for preliminary judgment of osteoporosis.

We found that the top five predictors were age, gender, disease history (DM, hypertension), and living habits (smoking). The results of Hiremath [28] uncovered that the risk increased by 20% with every 5 years increase of age. Besides, Fu et al. [29] believed that the most people aged 45–50 may suffer from osteoporosis, which is almost a common feature of human aging, and the incidence of a fracture depends on the age of the subject. Interestingly, some studies have demonstrated that postmenopausal women were susceptible to this disease, and low bone density had nothing to do with race [30, 31]. The main difference between the sexes is that males will produce larger bones than females as they grow, while females have better microstructures inside and have less chance of bone reconstruction. Furthermore, in direct or indirect mechanisms, sex hormones also play a leading and vital role in the physiology of bone [32]. Therefore, estrogen secretion in women during menopause is reduced, which ultimately leads to osteopenia and osteoporosis. Compared with women, men had a later age of bone loss. However, the mechanism between DM and bone condition was not fully understood clinically. In most studies, there was no consistent relationship between DM control and osteopenia in patients with type II DM [33]. Existing results shown that people with DM had lower bone density than people without DM. It can be concluded that there was an association between DM [34]. Besides, the available knowledge attested that smoking is a risk factor for osteoporosis [35, 36]. The latest study used national sample cohort data to conduct a retrospective study. The results showed that long-term abstinence from smoking reduced the risk of fracture in men [37].

This study used standard protocols and instruments, and all participants undergone complete health examinations. In order to ensure the collection of high-quality data, strict personnel training process was established. All these are the advantages, but the limitations also need to note. First of all, convenience sampling method may cause selection bias. Therefore, the generalization of the prediction model should be cautious. Secondly, the study was cross-sectional, not longitudinal. Some confounding factors cannot be eliminated in the research, causal association between low BMD and risk factors cannot be examined. Finally, the measurement of BMD at a certain point in time may be affected by survival effects.

## Conclusion

In summary, our study found that disease history and living habits were related to the risk of osteoporosis. The performance of the ANN model was better than other three models. The ANN model results showed that if the probability was more significant than 0.330, osteoporosis would occur. Further researches are needed to validate our model to predict the risk of osteoporosis in adults.

## Supplementary Information

**Additional file 1.**

## Data Availability

The datasets used and/or analyzed during the current study, are available from the corresponding author on reasonable request.

## Abbreviations

ANN: Artificial neural network
BMD: Bone mineral density
BMI: Body mass index
DBP: Diastolic blood pressure
SBP: Systolic blood pressure
CHD: Coronary heart disease
DM: Diabetes mellitus
WC: Waist circumference
SD: Standard deviation
OR (95% CI): Odds ratio with 95% confidence interval
Ref.: Reference
WHO: World health organization
ANOVA: Analysis of variance
ROC: Receiver operating characteristic
BP: Back propagation
AUCs: Area under receiver operating curves
DBN: Deep belief network
SVM: Support vector machine
GA-DT: Genetic algorithm - decision tree

## References

[1] Rozenberg S, Bruyère O, Bergmann P, Cavalier E, Gielen E, Goemaere S, Kaufman JM, Lapauw B, Laurent MR, de Schepper J, Body JJ (2020). How to manage osteoporosis before the age of 50. Maturitas.

[2] Lane NE (2006). Epidemiology, etiology, and diagnosis of osteoporosis. Am J Obstet Gynecol.

[3] Yu W, Wang R, Qu X. Regulating life-style & improving living habits can control Osteoporosis. Proceedings of the Third International Congress on Osteoporosis. 1999.

[4] Gregson CL, Newell F, Leo PJ, Clark GR, Paternoster L, Marshall M, Forgetta V, Morris JA, Ge B, Bao X, Duncan Bassett JH, Williams GR, Youlten SE, Croucher PI, Davey Smith G, Evans DM, Kemp JP, Brown MA, Tobias JH, Duncan EL (2018). Genome-wide association study of extreme high bone mass: contribution of common genetic variation to extreme BMD phenotypes and potential novel BMD-associated genes. Bone.

[5] Disse E, Ledoux S, Betry C, Caussy C, Maitrepierre C, Coupaye M, et al. An artificial neural network to predict resting energy expenditure in obesity. Clin Nutr. 2017;37. 10.1016/j.clnu.2017.07.017 28893410

[6] Shaabanpour F, Mollashahi B, Nosrati M, Moradi A, Sheikhpoor M, Movafagh A. Application of an artificial neural network in the diagnosis of chronic lymphocytic leukemia. Cureus. 2019;11(2):e4004. 10.7759/cureus.4004. 10.7759/cureus.4004 PMC6450593 31001458

[7] Eller-Vainicher C, Zhukouskaya V, Tolkachev Y, Koritko S, Cairoli E, Grossi E, Beck-Peccoz P, Chiodini I, Shepelkevich A (2011). Low bone mineral density and its predictors in type 1 diabetic patients evaluated by the classic statistics and artificial neural network analysis. Diabetes Care.

[8] Zeng J, Zhang J, Li Z, Li T, Li G. Prediction model of artificial neural network for the risk of hyperuricemia incorporating dietary risk factors in a Chinese adult study. Food Nutr Res. 2020;64:3712. 10.29219/fnr.v64.3712. 10.29219/fnr.v64.3712 PMC6983978 32047420

[9] Xu H, Wang Z, Li X, Fan M, Bao C, Yang R, Song F, Xu W, Qi X (2020). Osteoporosis and osteopenia among patients with type 2 diabetes aged ≥50: role of sex and clinical characteristics. J Clin Densitom.

[10] Risk WHO, Osteoporosis A (1994) Assessment of fracture Risk and its application to screening for postmenopausal Osteoporosis. Report of a WHO Study Group. 7941614

[11] Owen N, Leslie E, Salmon J, Fotheringham M (2000). Environmental determinants of physical activity and sedentary behavior. Exerc Sport Sci Rev.

[12] Zhu L, Dai X (2010). A study on physical fitness of professional young and middle-aged women with mental or manual labour. J Environ Occup Med.

[13] Hearty A, Gibney M (2009). Analysis of meal patterns with the use of supervised data mining techniques - artificial neural networks and decision trees. Am J Clin Nutr.

[14] Gupta H, Gupta P, Fang X, Miller W, Cemaj S, Forse R, Morrow L (2011). Development and validation of a Risk calculator predicting postoperative respiratory failure. Chest.

[15] O'Brien D, Sharkey Scott P. In: Chen H, editor. “Correlation and regression”, in approaches to quantitative research – a guide for dissertation students. Ireland. Oak Tree Press; 2012.

[16] Fawcett T (2006). Introduction to ROC analysis. Pattern Recogn Lett.

[17] Gholipour K, Asghari Jafarabadi M, Iezadi S, Janati A, Keshavarz S. Modelling the prevalence of diabetes mellitus risk factors based on artificial neural network and multiple regression. East Mediterr Health J. 2018;24(8):770–5. 10.26719/emhj.18.012. 10.26719/emhj.18.012 30328607

[18] Disse E, Ledoux S, Bétry C, Caussy C, Maitrepierre C, Coupaye M, Laville M, Simon C (2018). An artificial neural network to predict resting energy expenditure in obesity. Clin Nutr.

[19] Song J, Wu X, Zhang J, Zhang Y, Chen X (2017). Application of three statistical models in predicting the Risk of diabetes individuals. Chin J Health Statistics.

[20] Mukamal K, Ding E, Djoussé L (2006). Alcohol consumption, physical activity, and chronic disease risk factors: a population-based cross-sectional survey. BMC Public Health.

[21] Amato F, López-Rodríguez A, Peña-Méndez E, Vaňhara P, Hampl A, Havel J (2013). Artificial neural networks in medical diagnosis. J Appl Biomed.

[22] Li Z, Wu X, Gao X, Shan F, Ying X, Zhang Y, et al. Development and validation of an artificial neural network prognostic model after gastrectomy for gastric carcinoma: an international multicenter cohort study. Cancer Med. 2020;9(17):6205–15. 10.1002/cam4.3245. 10.1002/cam4.3245 PMC7476835 32666682

[23] Li H, Luo M, Zheng J, Luo J, Zeng R, Feng N, Du Q, Fang J (2017). An artificial neural network prediction model of congenital heart disease based on risk factors: a hospital-based case-control study. Medicine.

[24] Zhao Y, Zhao L, Mao T, Zhong L (2019). Assessment of risk based on variant pathways and establishment of an artificial neural network model of thyroid cancer. BMC Med Genet.

[25] Li Z, Li Y (2020). A comparative study on the prediction of the BP artificial neural network model and the ARIMA model in the incidence of AIDS. BMC Med Inform Decis Mak.

[26] Dey P, Lamba A, Kumari S, Marwaha N (2011). Application of an artificial neural network in the prognosis of chronic myeloid leukemia. Anal Quant Cytol Histol.

[27] Riedmiller M, Braun H (1993) A Direct Adaptive Method for Faster Backpropagation Learning: The RPROP Algorithm.

[28] Hiremath RN, Yadav AK, Ghodke S, Yadav J, Latwal S, Kotwal A (2018). Osteoporosis among household women: a growing but neglected phenomenon. Med J Armed Forces India.

[29] Fu G, Wang Y (1982). Aging and osteoporosis. Int J Geriatr.

[30] Sordia-Hernández L, Vazquez J, Iglesias JL, Piñeyro MO, Vidal O, Saldivar D, Morales A, Merino M, Pons G, Rosales E (2004). Low height and low weight correlates better with osteoporosis than low body mass index in postmenopausal women. Int Congr Ser.

[31] Becker C (2003). Clinical evaluation for osteoporosis. Clin Geriatr Med.

[32] Geusens P, Dinant G (2007). Integrating a gender dimension into Osteoporosis and fracture Risk research. Gender Med.

[33] Leidig-Bruckner G, Ziegler R (2001). Diabetes mellitus a risk for osteoporosis?. Exp Clin Endocrinol Diab.

[34] Mousa M, Elagrody A, Elhamed H, Hammad M. Relationship between diabetes mellitus and Osteoporosis. 2020.

[35] Silvennoinen JA, Lehtola J, Niemelä S (1996). Smoking is a Risk factor for Osteoporosis in women with inflammatory bowel disease. Scand J Gastroenterol.

[36] Rodionova SS, Khakimov UR, Morozov AK, Krivova AV. Smoking and alcohol abuse as risk factors causing low-energy fractures in males suffering from primary Osteoporosis. Health Risk Anal. 2020;(2):126–34. 10.21668/health.risk/2020.2.14.eng.

[37] Cho IY, Cho MH, Lee K, Park SM, Lee H, Son JS, Kim K, Choi S, Chang J, Koo HY, Bae YS, Kim SM (2020). Effects of smoking habit change on hospitalized fractures: a retrospective cohort study in a male population. Arch Osteoporos.

