# Supplementary material for: Prediction model for the risk of osteoporosis incorporating factors of disease history and living habits in physical examination of population in Chongqing, Southwest China: based on artificial neural network
Source: BMC Public Health. 2021 May 26;21:991. doi: 10.1186/s12889-021-11002-5 (PMC8157412; doi:10.1186/s12889-021-11002-5)
Supplement: Supplementary file 1 — Additional file 1. [file 12889_2021_11002_MOESM1_ESM.docx]

**Personal health status and lifestyle questionnaire**

**Part 1: General Information**

1. Your name__________

*2. Your gender

1. Male
2. Female

*3. Medical card number___________

**Part 2: Personal health**

**A. Do you have the following diseases or conditions? (single choice)**

*1. Hyperthyroidism

1. No
2. Yes, currently well controlled
3. Yes, currently poorly controlled

*2. Fracture

1. No
2. Yes, currently well controlled
3. Yes, currently poorly controlled

*3. Hypertension

1. No
2. Yes, currently well controlled
3. Yes, currently poorly controlled

*4. Coronary heart disease

1. No
2. Yes, currently well controlled
3. Yes, currently poorly controlled

*5. Diabetes mellitus

1. No
2. Yes, currently well controlled
3. Yes, currently poorly controlled

*6. Chronic gastrointestinal disease

1. No
2. Yes, currently well controlled
3. Yes, currently poorly controlled

*7. Chronic renal failure

1. No
2. Yes, currently well controlled
3. Yes, currently poorly controlled

*8. Gout

1. No
2. Yes, currently well controlled
3. Yes, currently poorly controlled

*9. Malignant tumor

1. No
2. Yes, currently well controlled
3. Yes, currently poorly controlled

*10. Corticosteroids (currently or previously used for more than three months)

1. No
2. Yes

*11. Currently taking anti-osteoporosis drugs

1. No
2. Yes

12. Other diseases (if any, please list)

**B. If you are a female, please answer the following questions (select each item or fill in the number)**

*1. The age of first menstruation is _ years old

*2. Whether you have menopause?

1. No
2. Yes, the menopausal age is _ years old

*3. Have you breastfed your child?

1. No
2. Yes, how many months of breastfeeding (__*)

*4. Are you taking estrogen drugs

1. No
2. Yes, how many years did you take it (__*)

**Part 3: Living habits**

**A. Smoke exposure**

*1. Do you smoke

- 1. Yes
  2. No

*2. Do you cook often (more than 5 days a week)

- 1. Yes
  2. No

**B. Drinking (select each item or fill in the number)**

*1. Do you drink

1. No
2. Yes

**C. Physical activity and physical exercise (select each item or fill in the number)**

*1. Nature of the work

1. sedentary
2. mild labor
3. manual labor

*2. The main modes of transportation to work

1. Working from home
2. Walking
3. Taking the bus
4. Driving

*3. Do the housework

1. Never
2. Occasionally
3. Often

Physical exercise

*1. In recent years, have you participated in physical exercises lasting more than 20 minutes each time?

1. Yes
2. No

*2. What is your usual physical exercise method?

1. Take a walk
2. Run
3. Swim
4. Ball games
5. Indoor fitness
6. Other

Note: Questions marked with * are required, otherwise the questionnaire cannot be submitted.
